# Manganese Ferrite Nanoparticles Encapsulated into Vitamin E/Sphingomyelin Nanoemulsions as Contrast Agents for High‐Sensitive Magnetic Resonance Imaging

**DOI:** 10.1002/adhm.202101019

**Published:** 2021-08-19

**Authors:** Sandra Díez‐Villares, Miguel A. Ramos‐Docampo, Andrés da Silva‐Candal, Pablo Hervella, Abi J. Vázquez‐Ríos, Ana B. Dávila‐Ibáñez, Rafael López‐López, Ramón Iglesias‐Rey, Verónica Salgueiriño, María de la Fuente

**Affiliations:** ^1^ Nano‐Oncology and Translational Therapeutics Group Health Research Institute of Santiago de Compostela (IDIS) SERGAS Santiago de Compostela 15706 Spain; ^2^ University of Santiago de Compostela (USC) Santiago de Compostela 15706 Spain; ^3^ Biomedical Research Networking Center on Oncology (CIBERONC) Madrid 28029 Spain; ^4^ Departamento de Física Aplicada Universidade de Vigo Vigo 36310 Spain; ^5^ CINBIO Universidade de Vigo Vigo 36310 Spain; ^6^ Clinical Neurosciences Research Laboratory Health Research Institute of Santiago de Compostela (IDIS) SERGAS Santiago de Compostela 15706 Spain; ^7^ Roche‐CHUS Joint‐Unit Translational Medical Oncology Group Health Research Institute of Santiago de Compostela (IDIS) SERGAS Santiago de Compostela 15706 Spain

**Keywords:** magnetic resonance imaging, nanoemulsions, sphingomyelin, superparamagnetic manganese‐ferrite nanoparticles, transverse relaxivity, vitamin E

## Abstract

Magnetic resonance imaging (MRI) is one of the most powerful non‐invasive imaging modalities used in clinics due to its great spatial resolution and excellent soft‐tissue contrast, though still less sensitive than other techniques such as the nuclear imaging modalities. This lack of sensitivity can be improved with the use of contrast agents based on nanomaterials. In recent years, researchers have focused on the development of magnetic nanoparticles, given their role as enhancers of the contrast signal based on the magnetic resonance. Manganese ferrite nanoparticles stand out, given their high magnetic susceptibility and magnetic soft nature. Herein, 10 nm MnFe_2_O_4_ nanoparticles, functionalized with the natural antioxidant vitamin E (VitE‐MFO) are encapsulated into simple, biodegradable and non‐toxic nanoemulsions (NEs), by a reproducible one‐step method obtaining stable 150 nm‐sized magnetic nanoemulsions (VitE‐MFO‐NEs). After encapsulation, the superparamagnetic properties of VitE‐MFO are maintained and MR imaging studies reveal an extremely high transverse relaxivity for VitE‐MFO‐NEs (652.9 × 10^−3^ m ^−1^ s^−1^), twofold higher than VitE‐MFO value. Moreover, VitE‐MFO‐NEs show great in vivo biocompatibility and good signal in in vivo and ex vivo MRI, which indicates their great potential for biomedical imaging enhancing the negative MR contrast and significantly improving the sensitivity of MRI.

## Graphical abstract

Vitamin‐E coated manganese ferrite nanoparticles are encapsulated into sphingolipid nanoemulsions by a one‐step method in order to provide reproducible and stable magnetic nanoemulsions. An extensive characterization is provided showing that these formulations maintain superparamagnetic properties, have a great in vivo biocompatibility and are able to improve the magnetic resonance imaging sensitivity, which is the main drawback of this technique.

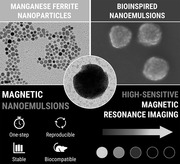

## 1 Introduction

The early detection of a disease plays a crucial role when it comes to its prognosis/diagnosis, especially in the case of oncological patients. Accordingly, there is an urgent need for more efficient tools for detailed and more accurate non‐invasive tissue and lesion imaging. Among the several non‐invasive imaging modalities currently used in clinic (magnetic resonance imaging (MRI), positron emission tomography (PET), computed tomography (CT) and ultrasound (US) imaging), MRI is one of the most used in clinical settings. This stems from the fact that it has great spatial resolution, being the most convenient technique to visualize pathological details at tissue depth, and despite its low sensitivity drawback.^[^ ^1^ ^]^ Nevertheless, the use of contrast agents in MRI significantly improves the sensitivity, such that more precise information of the disease region can be obtained as acquiring images with much higher quality.^[^ ^2^ ^]^

Currently, the contrast agents used in clinics for MRI are small molecules, mostly based on Gd‐based complexes.^[^ ^3^ ^]^ However, since this kind of molecules normally presents fast metabolism and non‐specific biodistribution through the body, they can also imply systemic toxic side effects. Alternatively, nanomaterials can be used to overcome the limitations of conventional contrast agents due to their unique properties of passive and active targeting, large payload ability, controlled engineering and the possibility to be combined with therapy for nanotheranostics applications.^[^ ^4^ ^]^ Considering these potential advantages of nanomaterials with respect to conventional contrast agents, the investigation in nano‐imaging agents has promptly increased in recent years. Moreover, besides the common function of anatomical MRI, contrast agents based on nanomaterials can also be exploited for functional MRI applications, such as the real‐time monitorization of the tumor temperature induced by photothermal therapy and the detection of specific molecule concentrations present in the tumor microenvironment.^[^ ^5^, ^6^ ^]^ Taking this into consideration, promising in vitro and preclinical results have been demonstrated, ending up with magnetic nanoparticles for example, already included in clinical programs.^[^ ^7^ ^]^

When it comes to magnetic nanoparticles, their magnetic moment is capable of inducing field inhomogeneities in the surrounding environment (typically composed by water molecules when the nanoparticles are immersed in tissue) when a radiofrequency pulse is applied. Therefore, magnetic nanoparticles show themselves as ideal candidates for enhanced MRI, owing to this strong interaction between their magnetic moment and the proton's magnetic moments in water molecules, which alters their relaxation and causes a different contrast resolution between different neighboring tissues. In this context, superparamagnetic nanoparticles of biocompatible spinel ferrites, such as manganese ferrite nanoparticles, with characteristic high values of saturation magnetization, become therefore appropriate for MRI applications, taking into account their T_2_‐weighted contrast signal.^[^ ^8^, ^9^, ^10^ ^]^

This outstanding potential shown by the magnetic nanoparticles can however become compromised, if taking into account their easiness to aggregate in different bio‐related media or their in vivo toxicity, demonstrated to produce cell oxidative stress that can lead to cell death.^[^ ^11^, ^12^ ^]^ While several surface coatings with different biocompatible compounds have been described to overcome these issues,^[^ ^13^, ^14^, ^15^, ^16^ ^]^ the encapsulation of the magnetic nanoparticles into organic nanoplatforms (e.g., liposomes, micelles, polymeric nanoparticles or nanoemulsions) can offer additional advantages. For example, besides enhancing their biocompatibility, their encapsulation can control the nanoparticles blood circulation time, ease the development of nanotheranostics strategies and even increase the sensitivity of MRI compared with free metal nanoparticles by enhancing the contrast signal.^[^ ^17^, ^18^, ^19^ ^]^ Lipid nanoemulsions can offer several advantages compared to other organic nanoplatforms. More in detail, these systems show higher loading capacity of lipophilic drugs and/or contrast agents due to their oily core and excellent colloidal stability due to the polar functional groups at their outer surface. These characteristics render them promising platforms for drug delivery, non‐invasive imaging and nanotheranostic applications.^[^ ^20^ ^]^ Furthermore, these lipid nanoemulsions can be tailored as well in terms of biocompatibility, taking into account biodegradable non‐toxic materials for their preparation.^[^ ^21^, ^22^ ^]^

Our group has recently reported the preparation and characterization of a bioinspired formulation based on lipid nanoemulsions using an one‐step, extremely simple and cost‐effective method.^[^ ^23^ ^]^ The main component is Vitamin E (VitE), which is a natural antioxidant and forms the oil droplets that are stabilized by sphingomyelin (SM), one of the main lipids of cell membranes. Previous results by our group highlight their high biocompatibility and versatility in nanomedicine, due to their capacity to carry hydrophobic drugs, biomolecules for gene therapy and radiometals for diagnosis applications.^[^ ^24^, ^25^ ^]^ Additional advantages of these nanosystems refer to their simplicity, great colloidal stability, and efficient internalization by the targeted cells.

With the above into account, we have reformulated our strategy, focusing in this study on the use of VitE/SM nanoemulsions (NEs) as lipid nanocarriers of manganese ferrite nanoparticles (MFO) with the aim of notably enhancing the sensitivity of the MRI technique. Here, we describe the synthesis and characterization of manganese ferrite nanoparticles coated and stabilized with VitE (VitE‐MFO) and their subsequent encapsulation in NEs to afford magnetic nanoemulsions (VitE‐MFO‐NEs). These magnetic nanocarriers were fully characterized in terms of their physicochemical, morphological and magnetic properties, providing evidences of the potential of these nanoplatforms as powerful MRI contrast agents.

## 2 Results and Discussion

**Figure 1:**
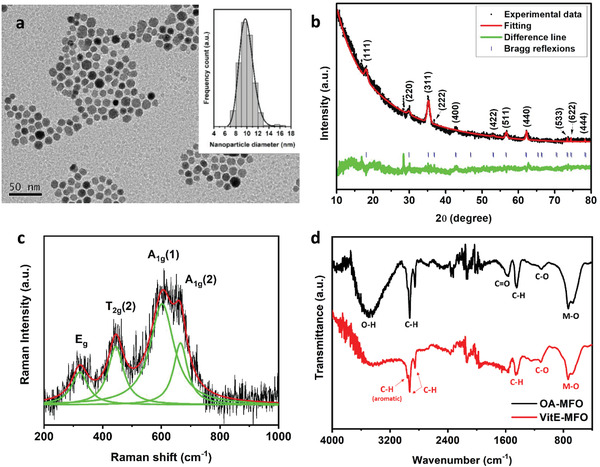
a) TEM image of the manganese ferrite nanoparticles, and the size histogram fitted to a log‐normal curve (inset). b) Experimental (black) and fitted (red) XRD pattern with the diffraction peaks indexed to the spinel structure. c) Raman spectrum registered using a 785 nm excitation wavelength with the deconvoluted vibrational modes (green). d) Infrared spectra of the oleic acid (black spectrum) and vitamin E (red spectrum) coated manganese ferrite nanoparticles.

Manganese ferrite nanoparticles (MFO) were obtained by a chemical thermodecomposition of the metal precursors (i.e., manganese and iron acetylacetonates) at high temperature. **Figure** **1** summarizes the morphological and structural characterization of the nanoparticles produced. The TEM image included in Figure 1a shows their well‐defined spherical shape, with an average diameter of 9.86*/1.02 nm (log‐normal fit). The structural characterization of the sample was performed using X‐ray diffraction (XRD) and Raman spectroscopy. The XRD pattern of the nanocrystals (Figure 1b) matches the expected Fd‐3m spatial group for the FCC spinel structure of the metal ferrite, with a Le Bail refinement offering a 0.84472 nm lattice parameter, which is in agreement with the expected for bulk manganese ferrite. Nevertheless, the inductively coupled plasma optical emission spectrometry (ICP‐OES) analysis revealed that the manganese ferrite is non‐stoichiometric (Mn_0.7_Fe_2.3_O_4_). Figure 1c includes the Raman spectrum of the sample, registered using a 785 nm excitation wavelength, at room temperature. The power was fixed below 0.5 mW, in order to avoid any structural transition in the sample.^[^ ^26^ ^]^ Group theory predicts five active Raman modes for the spinel structure, according to the *Fd‐3m* spatial group,^[^ ^27^ ^]^ namely A_1g_, 3T_2g_ and E_g_, though for nanoparticles only the A_1g_ (split), the E_g_ and one of the three T_2g_ are usually registered, as in this case (Lorentzial fit, in green). In this specific event of a non‐stoichiometric manganese ferrite, the splitting of the A_1g_ band can be associated to the presence of two different cations in tetrahedral positions (Mn^2+^ and Fe^3+^) according to the obtained stoichiometry. Accordingly, the three main bands observed in the spectrum, which can be related to the E_g_ (312 cm^−1^), the T_2g_(2) (450 cm^−1^) and the split A_1g_ (A_1g_(1) at 610 cm^−1^) and A_1g_(2) at 670 cm^−1^) vibrational modes, confirming the spinel ferrite crystalline structure. These bands also appear slightly shifted, likely because of the nanometric size of the crystals.

These magnetic nanoparticles are initially stabilized by an organic layer of oleic acid (OA) molecules attached to their surface, which keeps them stable in chloroform. In order to pave the way to encapsulate these MFO nanoparticles into the NEs, these OA molecules attached on the surface of the nanoparticles are first displaced/replaced by vitamin E (VitE), following the ligand exchange protocol detailed in the experimental section. Figure 1d includes FTIR spectra of the sample before and after promoting this ligand exchange at the surface of the nanoparticles. The black spectrum corresponds to the oleic acid coated nanoparticles, where the typical vibrational bands of oleic acid can be visualized, with the bands centered at 3496 cm^−1^ stemming from the O–H vibrational modes, and the bands located at 1113 and 1580 cm^−1^ from the C—O and C═O bonds respectively; all of them related to the carboxylic group of the oleic acid. In addition to those, the bands ranging from 2852 to 2962 cm^−1^ and the one centered at 1444 cm^−1^ correspond to the C–H stretching, or the C–H bending modes, respectively, of the hydrocarbon chain of the molecule. Alternatively, the red spectrum offers the vibrational modes after the ligand exchange. The disappearing of the stretching modes at 3496 and 1580 cm^−1^, associated to the carboxylic groups of the oleic acid just confirms the ligand exchange. Though bands centered at 1458 cm^−1^ and in the region ranging from 2854 to 2968 cm^−1^ are still present, they can be associated in this case to the C–H vibrational modes present in the vitamin molecule. The band at 1115 cm^−1^, which stems from the ether bond, can as well be associated to the vitamin, present in its molecule. Finally, it is necessary to highlight the presence of a broad band extended from 526 to 900 cm^−1^, which refers to the metal‐oxygen bond, typical of the inorganic core. Please, note that overtones at 2000–2200 cm^−1^ are related to the environmental carbon dioxide.

The selection of VitE for MFO coating was reinforced by the well‐known antioxidant activity of VitE which might help improve the biocompatibility of the magnetic nanoparticles by reducing their oxidant abilities, inhibiting or reducing the production of reactive oxygen (ROS) in vivo and thus, overcoming one of the main limitations for the translation of these nanoparticles to clinic.^[^ ^28^ ^]^ In addition, this previous coating of the nanoparticles with VitE renders easier the encapsulation in the Vitamin E‐ based nanoemulsions (vide infra).

**Figure 2:**
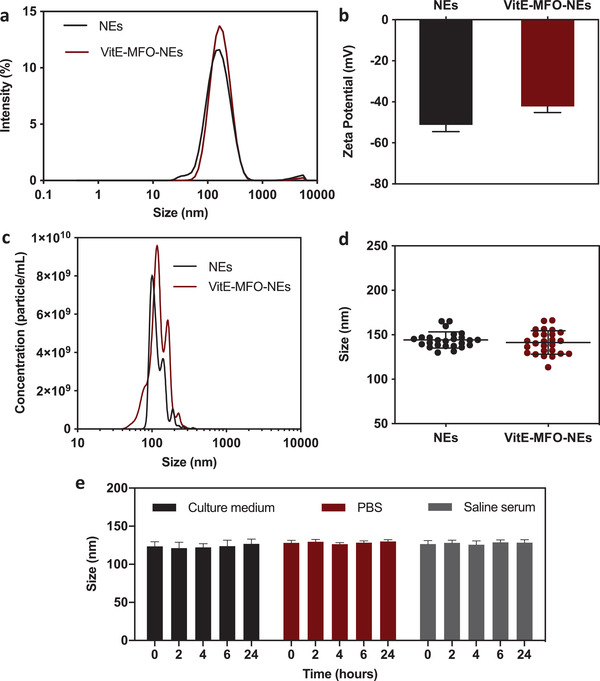
Physicochemical characterization of NEs and VitE‐MFO‐NEs by a) DLS, b) LDA, and c) NTA (*n* = 10). d) Reproducibility of the preparation method measuring the hydrodynamic size by DLS of 30 independent batches, horizontal bars represent mean and standard deviation (SD). e) Evolution of the average size of the VitE‐MFO‐NEs after incubation in DMEM culture medium, PBS 10 × 10^−3^ m pH 7.4 and saline serum (NaCl 0.9%), at 37 °C for 24 h (*n* = 5 per group).

In order to include the vitamin E‐coated MFO nanoparticles into the NEs, the organic phase composed by oil and surfactants (with or without VitE‐MFO) was injected in ultrapure water as shown in the scheme included in Figure S1 (Supporting Information). A homogeneous suspension of NEs and VitE‐MFO‐NEs (PDI = 0.19 in both cases) with an average size of 141 ± 3 nm or 149 ± 5 nm was spontaneously obtained after the injection, respectively, as shown by the DLS analysis (**Figure** **2a**). Figure 2b shows the *ζ*‐potential values of both NEs and VitE‐MFO‐NEs (−51 ± 3 and −42 ± 3 mV, respectively), ensuring the suspension stability due to electrostatic repulsions, analogously as previously reported for other magnetic emulsions.^[^ ^29^, ^30^, ^31^, ^32^ ^]^ The NEs and VitE‐MFO‐NEs were also characterized by tracking them using Nanoparticle Tracking Analysis (NTA), obtaining a mean size of 128 ± 39 and 136 ± 39 nm and concentrations of 4 × 10^11^ and 6 × 10^11^ NE per mL (Figure 2c). These results are consistent with the DLS analysis, but NTA offers better resolution than DLS allowing the observation of peaks very closed in size that cannot be detected by DLS.^[^ ^33^ ^]^ The results obtained from DLS and NTA show that the encapsulation of VitE‐MFO into the nanoemulsions did not produce significant variations in their physicochemical properties, giving similar size, zeta potential and concentration of both formulations. Thus, we have described the preparation of magnetic emulsions by an extremely simple and fast method. In fact, both formulations can be prepared in less than 10 min and in a very reproducible way as shown in Figure 2d. Furthermore, once prepared, colloidal stability studies of the VitE‐MFO‐NEs were assessed in three different relevant media (i.e., DMEM culture medium, phosphate buffer pH 7.4 and saline solution of NaCl 0.9%) by measuring the average size over time. The very slight variation in average size (within the error bar) (Figure 2e) in the three cases indicates the nanoemulsions keep the initial morphology over this period of 24 h at 37 °C in different media (and therefore exposed to different ionic strength or different biologically relevant content), demonstrating therefore their high stability, which is key for further in vitro and in vivo studies.^[^ ^34^ ^]^

Moreover, the magnetic nanoparticles encapsulation efficiency was determined by ICP‐OES after isolation and quantification of the free VitE‐MFO separated from the magnetic nanoemulsions, giving an encapsulation yield of 98% (Table S1, Supporting Information). Generally, the physicochemical properties of nanomaterials have been demonstrated to play a key role in the in vivo behavior of the formulation.^[^ ^35^ ^]^ For example, the mean size of nanomaterials is a key point in cancer applications as it strongly influences tumor accumulation and penetration; it is widely assumed that small nanoparticles can extravasate in the leaky vasculature of tumors due to the enhanced permeability and retention (EPR) effect,^36^ and that negative surface charge nanoparticles have longer blood circulation times and can reach the target organs more efficiently than positive ones.^[^ ^37^ ^]^

**Figure 3:**
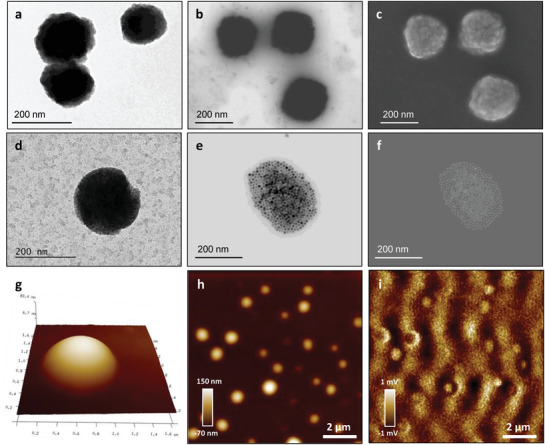
a) TEM image and FESEM images with b) STEM and c) InLens detectors showing the homogenous size distribution of NEs. Representative images showing the vesicular morphology of VitE‐MFO‐NEs acquired by d) TEM and e,f) FESEM. 3D‐high magnification image of the VitE‐MFO measured by g) AFM and general 2D topographic images of h) VitE‐MFO‐NEs in phase contrast and i) MFM modes, recorded in the same regions.

Structural characterization of NEs and VitE‐MFO‐NEs was performed by Field Emission Scanning Electron Microscopy (FESEM), Transmission Electron Microscopy (TEM), Atomic Forces Microscopy (AFM) and Magnetic Forces Microscopy (MFM), with representative images included in **Figure** **3**. Images of plain NEs were acquired by TEM (Figure 3a) and FESEM, using two detector modes, STEM (Figure 3b) and InLens (Figure 3c), reflecting the vesicular morphology and the homogeneous size distribution of the nanosystems. Characterization of the VitE‐MFO‐NEs was also performed, considering TEM, FESEM and also AFM, in order to correlate the 2D images with the three‐dimensional profile obtained by the atomic forces. Figure 3d,e,f include representative images of this partially organic, partially inorganic structure of the VitE‐MFO‐NEs formulation on which besides sphericity, we can appreciate the VitE‐MFO nanoparticles trapped inside NEs, without observing free magnetic cores. This is in agreement with the efficient entrapment already mentioned, such that the VitE‐MFO nanoparticles cannot freely diffuse in the outward direction through the lipidic shell. The AFM analysis, showed in Figure 3g, permits as well to appreciate the well‐defined spherical shape, preserved even after the NEs have been loaded with the VitE‐MFO nanoparticles (with a roundness factor of 0.92). AFM images plain NEs are shown in Figure S2 (Supporting Information). Additionally, AFM images using the magnetic force microscopy mode were carried out, in view of the magnetic response of the MFO nanoparticles encapsulated in the nanoemulsions. The MFM mode allows the study of magnetic forces at the nanoscale, by scanning the gradient of magnetic force on the sample surface while simultaneously obtaining the topographic map. Accordingly, Figure 3 includes a 10 × 10 µm^2^‐general overview of the VitE‐MFO‐NEs in phase contrast (Figure 3h) and MFM (Figure 3i) modes. Both images evidence the correlation between the NEs and the magnetic contrast stemming from the VitE‐MFO nanoparticles. In this regard, while the topography image (Figure 3h) shows the typical contrast between the sample and the substrate, and no phase contrast due to the magnetic particles, the MFM mode does exhibit a greater contrast because of the manganese ferrite in the presence of a 40 mT magnetic field (operating at 100 kHz with a lift height of 88 nm above the surface of the sample) over the same area (Figure 3i).

Indeed, the contrast varies gradually along with the scanning area, due to the overall response of the magnetic nanoparticles to the magnetized microscope tip (in this situation, black and white contrast refers to repulsive and attractive magnetic forces between the sample and the tip, respectively, as observed in similar analysis with magnetite and magnetite‐coated silica spheres).^[^ ^38^, ^39^ ^]^ Further investigations on the contrast signal of the MFM mode with the lift height were carried out, such that the images obtained (Figure S3, Supporting Information) reveal that the magnetic signal faints with increasing the lift height. This stems from the fact that magnetic interactions are distance dependent, and accordingly, the larger the lift height the weaker the magnetic interactions established between the nanoparticles and the tip of the MFM mode, effect not implied when considering the phase contrast signal. This scenario just reinforces the efficient encapsulation of magnetic VitE‐MFO nanoparticles into the NEs, as previously discussed.

**Figure 4:**
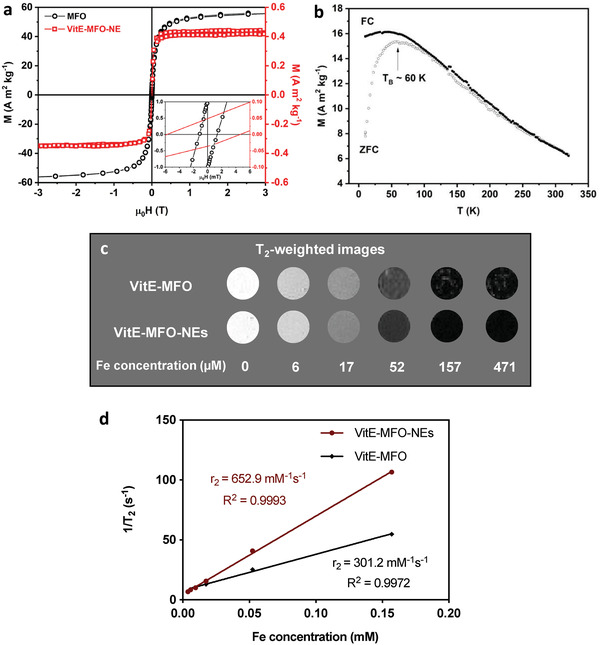
a) Magnetization (M) versus magnetic field (µ0H) at room temperature of the manganese ferrite nanoparticles (black) and VitE‐MFO‐NEs at 300 K (red). b) ZFC/FC curves of the manganese ferrite nanoparticles. c) T2‐weighted images and d) transverse relaxivity of VitE‐MFO and VitE‐MFO‐NEs acquired with 9.4 T horizontal MR scanner.

The magnetic characterization of the free VitE‐MFO nanoparticles before and after encapsulation into the NEs (VitE‐MFO‐NEs) is included in **Figure** **4**. Figure 4a (black) includes the hysteresis loop of the sample of nanoparticles at 300 K), reflecting the very small coercivity approaching zero at room temperature. This is generally associated to the superparamagnetic behavior of nanoparticles with a very small magnetocrystalline anisotropy in agreement with the soft nature of the ferrimagnetic manganese ferrite.^[^ ^40^ ^]^ The value of saturation magnetization (*M_S_*) was found to be 57.0 Am^2^ kg^−1^ at room temperature, which are smaller than the tabulated value of the bulk manganese ferrite, in agreement with the non‐stoichiometry already mentioned.^[^ ^16^, ^18^, ^41^ ^]^ The field‐dependent magnetization hysteresis loop at 300 K of the magnetic VitE‐MFO nanoparticles once trapped inside the NEs is also included in Figure 4a (in red), confirming the superparamagnetic behavior of the manganese ferrite nanoparticles after their encapsulation into NEs, and showing a decrease in the value of magnetization per mass of sample, given the rather large weight percentage of the diamagnetic organic lipid nanoemulsions.^[^ ^42^, ^43^ ^]^ This also indicates there is no structural or chemical degradation of the manganese ferrite magnetic material after the entrapment process, rendering therefore the magnetic NEs suitable for MRI applications. Furthermore, the ZFC/FC temperature‐dependent magnetization curves reflect the superparamagnetic behavior of the nanoparticles employed, (Figure 4b), exhibiting a blocking temperature (*T*_B_) at very low temperature (≈60 K). The absence of a flat plateau below *T*_B_ in the FC curve is a clear indicator that no strong dipolar interactions are taken place between nanoparticles, even in the powdered samples. It should be noted that the blocking temperature of the nanoparticles is located at a relatively high temperatures as the measurement is performed with the sample as compacted powder, and therefore, with the nanoparticles strongly interacting. In case of having the nanoparticles far apart, this temperature would decrease. In the case of the hysteresis loops, the coercivity value in the field dependent magnetization implies a strongly interacting system. When shifting from the sample of nanoparticles as compact powder to the sample of nanoparticles grouped in the NEs, the system becomes less dipolar interacting but the coercivity does not notably change, as shown in Figure 4a, meaning that the nanoparticles are still interacting.

Then, to demonstrate the potential of the developed VitE‐MFO‐NEs as negative MRI contrast agents, we have evaluated the magnetic sensitivity using 9.4 T horizontal MR scanner. Agar‐based phantom with different concentrations of VitE‐MFO and VitE‐MFO‐NEs were considered to study the dependence of the MR signal intensity on the metal concentrations indicated. Figure 4c shows a sequence of T_2_‐weighted images on which we can appreciate the gradual enhancement of the negative contrast signal when the iron concentration is increased. This means that indeed the MFO nanoparticles can shorten the transversal relaxation time of water protons (T_2_) and can therefore be efficiently used as negative MR contrast agents.

Moreover, the transverse relaxivity (r_2_) values resulting from the slope of linear fit between the inverse of transversal relaxation times (1/T_2_) versus iron concentrations (´10 ^−3^ m Fe) are shown in Figure 4d. Free VitE‐MFO (dispersed in chloroform) showed high relaxivity values (301.2 ´ 10 ^−3^ m ^−1^ s^−1^), suitable for T_2_ enhanced MRI. The high relaxivity measured for VitE‐MFO was even improved after encapsulation in VitE‐MFO‐NEs, whose r_2_ value turned out to be twofold higher than VitE‐MFO (652.9 ´ 10 ^−3^ m ^−1^ s^−1^). The obtained r_2_ values are somehow expected since manganese‐doped ferrite nanoparticles have been reported as greater enhancers of the MRI signal compared to conventional iron oxide nanoparticles.^[^ ^8^, ^9^, ^10^ ^]^ Further, we could highlight that the values obtained in this report are sixfold higher than ferumoxytol (r_2_ value of 98.4 ´ 10 ^−3^ m ^−1^ s^−1^ at 7.0 T), which is currently the only FDA‐approved iron oxide formulation used as off‐label MRI contrast agent.^[^ ^41^, ^44^ ^]^ We also investigated their potential as dual contrast agents and so, the longitudinal relaxivity (r_1_) of VitE‐MFO‐NEs was calculated (Figure S4, Supporting Information). The measured r_1_ value was very low (0.653 ´ 10 ^−3^ m ^−1^ s^−1^), which is in agreement with other systems measured at high magnetic fields.^[^ ^45^ ^]^ This just implies that VitE‐MFO can be used as strong T_2_ contrast agents at 9.4 T field, but cannot work as T_1_ contrast agent. While the transverse relaxivity values of nanoparticles are directly dependent on magnetic saturation magnetization the theoretical models accounting for the transverse relaxation of magnetic materials rely on the fact that particles can be subjected to the motional average regime (MAR, if Δ*ωτ*_D_ < 1) or be in the static dephasing regime (SDR, if Δ*ωτ*_D_ > 1). The factor Δ*ω* *τ*_D_ =  *γμ*_0_ *M*_v_ *d* ^2^/12*D* accounts for the role that diffusion plays in the nuclear magnetic resonance decay, being negligible for larger particles. Roughly speaking, small particles, or particles immerse in micelles or inside porous materials (or with an overall magnetic moment reduced compared to the pure magnetic core) are governed by the MAR. In this case, both VitE‐MFO and VitE‐MFO‐NE are found to be in the MAR, and hence their transverse relaxivity can be expressed as:

(1) $$r_{2}=\frac{4\gamma^{2}\mu_{0}^{2}v_{\mathrm{mag}}M_{v}^{2}d^{2}}{405\;D}\;$$

where *γ* stands for the proton gyromagnetic ratio (42.6 MHz T^−1^), *µ_0_* for the magnetic permeability of vacuum (4*π* × 10^−7^ T m A^−1^), *v*_mag_ is the molar volume of the magnetic material, *M*_v_ is the volumetric magnetic saturation, *d* is the diameter of the nanoparticle and *D* is the diffusion coefficient of water (3 × 10^−9^ m^2^ s^−1^). Thus, according to equation (1), a lower value of r_2_ can be expected for VitE‐MFO‐NEs, considering the lower magnetic saturation they display if compared to the free MFO nanoparticles. However, within the nanoemulsions the magnetic nanoparticles can establish strong dipolar interactions, that can be therefore related to the high r_2_ values registered.^[^ ^18^, ^46^, ^47^ ^]^

**Figure 5:**
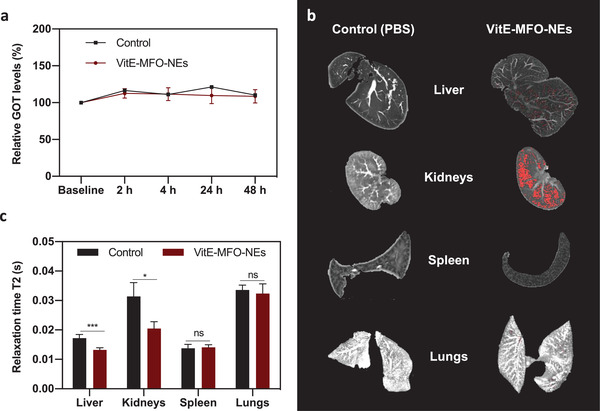
a) GOT levels normalized to baseline at different time points after the injection of VitE‐MFO‐NEs (*n* = 4) and PBS (control). Representative ex vivo T_2_‐maps MR images of rat organs 48 h after the intravenously injection of PBS (left) and VitE‐MFO‐NEs (right). b) Hyposignal caused by VitE‐MFO‐NEs are label in red. c) Ex vivo biodistribution of VitE‐MFO‐NEs (*n* = 4) compared to control (PBS) determined by the relaxation times. * (*p* < 0.05), ** (0.05 >  *p* < 0.001), *** (*p* < 0.0001) was considered statistically significant.

With the aim to investigate the in vivo MR applications of VitE‐MFO‐NEs we first evaluated their in vivo biocompatibility in Sprague‐Dawley rats by measuring biological parameters that can indicate the safety/toxicity of these nanosystems. It is well known that most of the nanoparticles are sequestered by the liver after intravenous administration and thus, it is the main organ that can be damaged due to nanoparticle accumulation.^[^ ^48^ ^]^ In addition, kidneys also play a crucial role in the accumulation of metabolized nanoparticles before their elimination through the urine. In this sense, we have injected VitE‐MFO‐NEs intravenously (1 mL, 471 × 10^−6^ m Fe, 1.88 µmol Fe kg^−1^, *n* = 4) and PBS pH 7.4 as control in healthy rats, and measured the blood levels of Glutamate Oxaloacetate Transaminase (GOT) and creatinine at different time points, as biomarkers for hepatic and renal damage, respectively. An increase in GOT levels (with respect to the animal's baseline levels) would be indicative of hepatotoxicity, as well as increased creatinine levels would be related to nephrotoxicity. As shown in **Figure** **5a** no differences in GOT levels were found with respect to the control animal in all the time points studied. Moreover, creatinine levels were in all cases under the detection limit of the technique (<0.5 mg dL^−1^), indicating that VitE‐MFO‐NEs do not provoke any hepatic or renal toxicity at the studied dose. These results are in line with our previous work where we demonstrate the biocompatibility of VitE‐NEs in different cell culture lines, zebrafish and mice.^[^ ^23^ ^]^ After 48 h post‐administration, animals (*n* = 4) were perfused transcardially and organs were removed from the body to determine the VitE‐MFO‐NEs ex vivo biodistribution by MRI. Liver, lungs, kidneys and spleen were placed in agar‐based phantoms and T_2_‐weighted images were acquired with 9.4 T horizontal MR scanner. Figure 5b shows T_2_‐maps of animal's organs calculated from T_2_‐weighted images. To quantify the hyposignal caused by VitE‐MFO‐NEs, T_2_ relaxation times of the control organs (from rats injected with PBS) were measured and a mean of the minimum values were selected as a threshold, which was applied to the treated animals. Relaxation times lower than the selected threshold indicate the presence of VitE‐MFO‐NEs in the organs and these pixels were marked in red in Figure 5b. As shown in Figure 5b,c, VitE‐MFO‐NEs are accumulated mainly in liver and kidneys 48 h post‐administration, in agreement with other NEs described for this applications.^[^ ^30^, ^31^, ^49^ ^]^ It is also noteworthy the absence of nanoparticles in lungs, which could be an indicative that the VitE‐MFO‐NEs are stable and do not aggregate causing obstruction in the pulmonary capillaries. Therefore, our results indicate that VitE‐MFO‐NEs are biocompatible and can be detected by ex vivo MRI at extremely low doses related to their high transverse relaxivity.^[^ ^50^ ^]^

**Figure 6:**
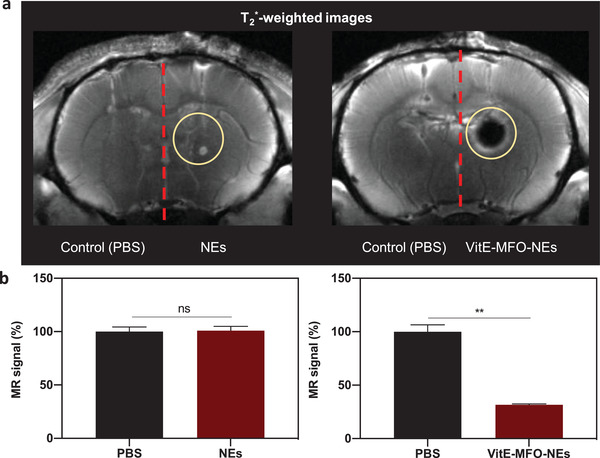
a) T2*‐weighted MR images of brain rats injected in the left cerebral hemisphere with PBS as a control and in the right hemisphere with the formulations NEs (image on the left) and VitE‐MFO‐NEs (image on the right). Comparison of T2*‐weighted MR signals between PBS and NEs (left) and PBS and VitE‐MFO‐NEs (right). b) MR signals of NEs and VitE‐MFO‐NEs are normalized to PBS as control (100%). (*n* = 3 per group) * (*p* < 0.05), ** (0.05 > *p* < 0.001), *** (*p* < 0.0001) was considered statistically significant.

Apart from whole‐body imaging applications, MRI is one of the preferred techniques in the diagnosis of neurological pathologies due to its high spatiotemporal resolution and unlimited tissue penetration depth, allowing the use of multiple sequences to visualize and evaluate the functionality and structure of different brain regions.^[^ ^51^, ^52^ ^]^ For this reason, we also have performed a proof of concept in vivo study to evaluate the potential application of the VitE‐MFO‐NE as contrast agents in the brain. NEs and VitE‐MFO‐NEs were injected in the right hemisphere of the brain (10 µL, 471 × 10^−6^ m Fe, 0.018 µmol Fe kg^−1^, *n* = 3), and as a control, PBS was injected in the left hemisphere (*n* = 3). **Figure** **6** shows the strongly negative contrast of the developed magnetic nanoemulsions after intracranial injections. T_2_^*^‐weighted in vivo MR images of rat brains (Figure 6a) and graphics of the relative MR signal measured (Figure 6b) show the efficient negative contrast produced by VitE‐MFO‐NEs compared with PBS and plain NEs. Similar results were obtained with T_2_‐weighted images and are shown in Figure S5 (Supporting Information). Overall, we have proved that VitE‐MFO‐NEs can dramatically enhance the T_2_^*^‐weighted and T_2_‐weighted signals, offering therefore efficient formulations for high‐sensitive MRI. Further experiments will involve testing of these formulation in relevant animal models so they will allow us to determine their full potential as high‐sensitive tissue MR imaging.

## 3 Conclusion

We have successfully prepared a very simple, easy to manufacture and biocompatible formulation for high‐sensitive T_2_ MRI by a one‐step method. First, manganese ferrite nanoparticles were successfully synthesized by thermodecomposition of the metal precursors and coated for the first time with the antioxidant Vitamin E to obtain hydrophobic nanoparticles. Then, VitE‐MFO were encapsulated into biocompatible VitE/SM NEs providing good‐sized, homogeneous, negative charged and highly stable formulations suitable for biomedical applications. Moreover, morphological characterization of VitE‐MFO‐NEs confirmed the narrow distribution of the particles and the efficient entrapment of VitE‐MFO into their inner core. We have demonstrated the magnetic properties of VitE‐MFO‐NEs by confirming that the superparamagnetic behavior of the nanoparticles was not compromised after encapsulation. In vitro MR studies of VitE‐MFO and VitE‐MFO‐NEs showed a great enhancement of the T_2_‐weighted MR contrast, being significantly higher for VitE‐MFO‐NEs. Moreover, we have proved the in vivo biocompatibility of VitE‐MFO‐NEs and confirmed that they can be detected by ex vivo MRI and in vivo MR brain imaging. In summary, this work provides an exhaustive characterization of biocompatible magnetic nanoemulsions that possess an ultrahigh T_2_ contrast ability and can efficiently increase the sensitivity or MRI and thus, open a gate for possible applications in biomedical imaging.

## 4 Experimental Section

### Materials

Manganese(II) acetylacetonate (Mn(acac)_2_, 98%), iron(III) acetylacetonate (Fe(acac)_3_, 98%), (±)‐*α*‐tocopherol (96%, VitE) used for the coating of the magnetic nanoparticles, folic acid (>97%, FA), oleylamine (70%), benzyl ether (98%) and agar were purchased from Sigma‐Aldrich. MilliQ water (Millipore) was used through the study (18.2 MΩ cm resistance). Sphingomyelin was purchased from Lipoid GmbH. The surfactant C16/C18‐COO‐C_9_H_9_O_3_ was synthesized by GalChimia. Solvents used such as absolute ethanol and chloroform, were acquired from Cienytech and Thermo Fisher Scientific. All other chemicals and reagents were of analytical grade.

### Synthesis of Manganese Ferrite Nanoparticles (MFO)

The nanoparticles were synthesized by the chemical thermodecomposition of the manganese and iron metal precursors, following a procedure published elsewhere.^[^ ^53^ ^]^ Briefly, Fe(acac)_3_ (1.33 mmol), Mn(acac)_2_ (0.67 mmol), 1,2‐dodecanediol (10 mmol), OA (6 mmol), oleylamine (6 mmol), and benzyl ether (20 mL) were mixed and magnetically stirred. The mixture was heated to reflux up to 200 °C for 2 h and then, heated up to 300 °C for 1 h. The black‐colored mixture was allowed to cool down to room temperature, and the colloids were washed three times in ethanol by centrifugation and dried by letting the solvent evaporate. These nuclei (84 mg) were used as seeds, repeating the previous protocol, in order to have a second step of growth to attain 10 nm average diameter manganese ferrite nanoparticles. In this second step, the mixture was first heated to reflux up to 200 °C for 1 h, and then heated up to 300 °C for 30 min. Afterwards, colloids were washed in ethanol by centrifugation and dispersed in chloroform while adding oleic acid (0.8 mmol into a 5 mL solution of the MnFe_2_O_4_ particles, 2 mg mL^−1^). An additional step by which exchanging the oleic acid molecules by (±)‐*α*‐tocopherol was carried out. For that, 5 mL of the previous solution of the manganese ferrite nanoparticles (2 mg mL^−1^) were first transferred into 5 mL of cyclohexane including 1 mmol of *α*‐tocopherol, mixed and stirred at room temperature for 2 h, to promote the ligand exchange. The solution was further diluted, up to 10 mL of cyclohexane, and heated at 60 °C for 1 h. Finally, the colloids were washed with a mixture of ethanol/cyclohexane by centrifugation. The resultant product was dispersed in chloroform, with a concentration of 2 mg mL^−1^ (referred to the inorganic core).

### Characterization of MFO

Transmission electron microscopy measurements were performed on a JEOL JEM 1010 instrument operating at an acceleration voltage of 100 kV. Nanoparticles for the TEM analysis were prepared by dropping a diluted suspension of the sample onto an ultrathin carbon‐coated copper grid. X‐ray diffraction (XRD) patterns were collected by using a PANalytical X'Pert PRO diffractometer (CuK_a_ radiation, *λ* = 1.54056 Å)). Raman spectra were collected with a Renishaw in Via Reflex Raman Microscope. Experiments were conducted at room temperature by using an excitation wavelength of 785 nm. ICP‐OES measurements were conducted by treating the samples with nitric acid (2 vol%) while heated at 120 °C and analyzed in an Optima 4300DV (PerKin Elmer). Magnetic measurements of VitE‐MFO and VitE‐MFO‐NEs were performed by using a Physical Property Measurement System (PPMS) from Quantum Design. Hysteresis loops were measured at 10 and 300 K applying a magnetic field up to 5 T (or up to 3 T for VitE‐MFO samples). The temperature‐dependent magnetization in zero‐field‐cooling (ZFC) and field‐cooling (FC) conditions was performed at 10 mT in the 2–330 K range. The magnetic measurements were performed using dried powdered samples.

### Formulation of NEs and VitE‐MFO‐NEs

Oil in water (O/W) nanoemulsions were prepared by ethanol injection. Briefly, vitamin E (5 mg), sphingomyelin (0.5 mg) and C_16_/C_18_‐COO‐C_9_H_9_O_3_ (0.5 mg) were dissolved in 100 µL of absolute ethanol with the lipid ratio 1:0.1:0.1 w/w. The organic phase was injected in 1 mL of MilliQ water using an insulin syringe (0.5 mL, 0.33 × 12 mm ICO.C.1) under magnetic stirring giving a total lipid concentration of 5.5 mg mL^−1^. The encapsulation of manganese ferrite nanoparticles coated with vitamin E (VitE‐MFO) into the nanoemulsions was performed by the addition of 120 µg of VitE‐MFO dissolved in chloroform (12 mg mL^−1^) to the organic phase with the lipids suspended in ethanol. This phase was sonicated for 5 min in an ice bath and injected with an insulin syringe into water. The suspension was kept under orbital agitation for 10 min at room temperature to reduce the residual traces of chloroform. Based on their hydrophobicity, non‐encapsulated nanoparticles precipitate in aqueous medium after centrifugation and they were removed by collecting the supernatant and discarding the precipitate.

### Physicochemical Characterization

NEs and VitE‐MFO‐NEs were characterized in terms of their hydrodynamic diameter and correspondent size distribution, which is defined by the polydispersity index (PDI), by Dynamic Light Scattering (DLS) using a Nanosizer 2000 (Malvern Instruments). The measurements were made by diluting samples in MilliQ water (dilution 1:10) in disposable microcuvettes (ZEN0040, Brand). The zeta potential (ZP) was measured by laser Doppler anemometry (LDA). To do the measurement, a dilution 1:40 of the nanoemulsions in MilliQ water was disposed in folded capillary cells cuvettes (DTS 1070, Malvern Instruments). NEs and VitE‐MFO‐NEs were also characterized by Nanoparticle Tracking Analysis (NTA) diluting the particles 1:1000 in MilliQ water (NanoSight LM20). Data were collected with 3 captures of 60 s and both shutter and gain were manually determined for each sample. TA 2.0 Build 127 software was used for measurement and subsequent data analysis. For stability studies, size and PDI of the nanoemulsions were measured along time after incubation of the particles in different media at 37 °C: Dulbecco's modified Eagle's culture medium (DMEM, Merck Group), saline solution (sodium chloride, NaCl 0.9%) and phosphate buffer (PBS, 10 × 10^−3^ m). All the formulations and measurements were performed at least in triplicate.

### Morphological Characterization

The morphology of the NEs and VitE‐MFO‐NEs was observed by Transmission Electron Microscopy (TEM), Field Emission Scanning Electron Microscopy (FESEM) and Atomic Forces Microscopy (AFM). For NEs, 20 µL of nanoemulsions (0.5 mg mL^−1^) were stained with 20 µL phosphotungstic acid (2% w/v) and placed on a carbon coated grid. The excess was washed with MilliQ water and the grid was allowed to dry overnight at room temperature. FESEM images were acquired using a ZEISS FESEM ULTRA Plus, microscope with two detectors, STEM, and InLens. Characterization of VitE‐MFO‐NEs was done by the same method described above for free MFO. AFM characterization was performed in a Scanning Probe Microscopy Multimode Multimode 8/Nanoscope V operating in tapping mode. The MFM mode was employed using a Tip Roc < 35 nm (radius) with a CoCr coating, working at a nominal frequency of 75–100 kHz and a nominal coercivity of 40 mT. Samples for AFM/MFM were prepared in Millipore water and allowed to dry over a mica substrate overnight at room temperature.

### In Vitro MR Imaging

MR imaging was performed by agar‐based phantoms prepared following a procedure described elsewhere.^[^ ^54^ ^]^ Agar solution (1.6% w/v) was used to prepare a mold with several wells. Different dilutions of the nanoemulsions were prepared in order to obtain serial concentrations of manganese ferrite nanoparticles ranging from 0.006 to 0.471 × 10^−3^ m Fe. Then, 100 µL of magnetic nanocarriers were mixed with 100 µL of agar solution, deposited into agar wells and covered with the remaining agar solution. For VitE‐MFO phantoms dilutions were made in ethanol and then mixed with agar solution. MR images were acquired on a Bruker Biospec 9.4 T small animal MR scanner (horizontal bore magnet with 12 cm wide Bruker BioSpin) equipped with actively shielded gradients (440 mT m^−1^). For the acquisition of data, a radiofrequency resonator (Bruker) was used as transmitter‐receiver (quadrature volume coil 7 cm in diameter).

T_2_‐weighted images were acquired using the multi‐slice multi‐spin‐echo (MSME) sequence with 10.26 ms of echo time, 3 s of repetition time, 16 echoes with 10.26 ms echo spacing, 50 kHz spectral bandwidth, flip angle of 90 °, 14 slices of 1 mm, 1 average. T_2_‐weighted images were obtained with a field of view (FOV) of 7.5 × 7.5 cm (with saturation bands to suppress signal outside this FOV), and a matrix size of 300 × 300, giving an in‐plane resolution of 250 µm/pixel and implemented without fat suppression. Images were processed, and their T_2_‐maps were calculated using the software FIJI: Image J (Rasband W, NIH). The relaxivity constant (r_2_) was calculated as the slope of the curve obtained by fitting the T_2_^−1^ values versus the Fe concentration in ×10^−3^ m.

### In Vivo Studies

Animal experiments were conducted in the Clinical Neurosciences Research Laboratory of the University Clinical Hospital of Santiago de Compostela (REGA ES 15078 029 2801). All experimental animal procedures were conducted under the procedure number: 15010/2019/004 approved by the Animal Care Committee, according to European Union Rules and the Spanish regulation (86/609/CEE, 2003/65/CE, 2010/63/EU, RD1201/2005 and RD53/2013, RD 53/2013). Sprague‐Dawley rats with a weight between 250 and 300 g were used for in vivo studies. In all the experiments animals were anesthetized placing them in a stereotaxic frame (Stoelting Co., Wood Dale) under sevoflurane anesthesia.

### In Vivo Biocompatibility of VitE‐MFO‐NEs

VitE‐MFO‐NEs (*n* = 4, 2.7 mg lipids mL^−1^, 471 × 10^−6^ m Fe) were injected through the tail vein in anesthetized rats (1 mL, 10.9 mg lipids kg^−1^, 1.88 µmol Fe kg^−1^) and hepatic and renal toxicities were assessed measuring Glutamate Oxaloacetate Transaminase enzyme (GOT) and Creatinine levels at different time points (prior injection and 2, 4, 24, 48 h post‐injection). The same volume of PBS was intravenously injected as a control. At the selected time points, blood was extracted from the tail vain and collected in tubes with heparin (BD Vacutainer Heparin Blood Collection Tubes). Then, 32 µL of blood were placed in reactive strips for GOT (ref: 10745120202 Roche), and creatinine (ref: 10886874202 Roche) and analyzed in a Reflotron plus (Roche, Basel, Switzerland). GOT levels were normalized to the baseline levels of each animal.

### Ex Vivo MRI

After 48 h from PBS/VitE‐MFO‐NEs intravenous injection (*n* = 4), animals were anesthetized and perfused transcardially with 100 mL PBS 0.1 M pH 7.4 and 150 mL 4% formaldehyde (VWR Chemicals, Leuven, Belgium). Organs (lungs, kidneys, liver and spleen) were carefully removed and postfixed by immersion in 4% formaldehyde until MRI analysis. Agar‐based phantoms were prepared with these organs in order to determine T_2_ relaxation times. MR images were acquired following the protocol previously described for in vitro MRI studies and T_2_‐maps were calculated from T_2_‐weighted images. Image analysis was performed selecting each control organ as a region of interest (ROI) and measuring a mean of the minimum relaxation times values. These values were fixed as threshold and were applied to the organs treated with VitE‐MFO‐NEs in order to quantify the differences caused by these particles in the relaxation times.

### In Vivo Brain MRI

For brain MR imaging VitE‐MFO‐NEs, VitE‐NEs and PBS were injected directly in the parenchyma of the animals (*n* = 3, 2.7 mg lipids mL^−1^, 471 × 10^−6^ m Fe) following a protocol previously described.^[^ ^55^ ^]^ Briefly, a Hamilton syringe (Hamilton; 10 µL) was filled with the corresponding nanoparticle suspension, NEs (0.109 mg lipids kg^−1^) or VitE‐MFO‐NEs, (0.109 mg lipids kg^−1^, 0.018 µmol Fe kg^−1^) and a volume of 10 µL was injected in the right hemisphere of the brain at a flow rate of 1 µL min^−1^ over 10 min. Same procedure was performed in the left hemisphere injecting 10 µL of PBS. The rats were placed in an animal box after surgery for recovering in a warm place with access to food.

MRI studies were conducted on a 9.4‐T horizontal bore magnet (Bruker BioSpin) with 12‐cm wide actively shielded gradient coils (440 mT m^−1^), and a combination of a linear birdcage resonator (7 cm in diameter) for signal transmission and a 2 × 2 surface coil array for signal detection, positioned over the head of the animal, which was fixed with a teeth bar, earplugs, and adhesive tape. Transmission and reception coils were actively decoupled from each other. Gradient‐echo pilot scans were performed at the beginning of each imaging session for accurate positioning of the animal inside the magnet bore. In order to assess nanoparticles in vivo, animals were scanned following T_2_‐weighted and T_2_*‐weighted sequences in MRI to evaluate the presence and the distribution 1 h after the injection. T_2_*‐weighted images were acquired using a MGE sequence with a 2.9 ms echo time, 1.5 s repetition time, 16 echoes with 3.28 ms echo spacing, implemented with a FA of 30°, 2 averages, 14 slices of 1 mm, and with a 19.2 × 19.2 mm^2^ FOV, a 192 × 192 image matrix (isotropic in‐plane resolution of 100 µm pixel^−1^). T_2_‐weighted images were acquired using a MSME sequence with a 9 ms echo time, 3 s repetition time, 16 echoes with 9 ms echo spacing, implemented with a FA of 180°, 2 averages, 14 slices of 1 mm and 19.2 × 19.2 mm^2^ FOV, a 192 × 192 image matrix (isotropic in‐plane resolution of 100 µm pixel^−1^). MRI post‐processing was performed using ImageJ software (W. Rasband, NIH, USA).

### Statistical Analysis

All the experiments were performed at least in triplicate. Data are expressed as mean ± standard deviation (SD). Statistical analyses were calculated with GraphPad Prism software (version 8.0). Student's *t*‐test was used to compare significant differences between two groups. * (*p* < 0.05), ** (0.05 > *p* < 0.001), *** (*p* < 0.0001) was considered statistically significant.

## Conflict of Interest

The authors declare no conflict of interest.

## Supporting information

Supporting Information

## Data Availability

Data available on request from the authors.
