# Supplementary material for: Manganese Ferrite Nanoparticles Encapsulated into Vitamin E/Sphingomyelin Nanoemulsions as Contrast Agents for High‐Sensitive Magnetic Resonance Imaging
Source: Adv Healthc Mater. 2021 Aug 19;10(21):2101019. doi: 10.1002/adhm.202101019 (PMC11469163; doi:10.1002/adhm.202101019)
Supplement: Supplementary file 1 — Supporting Information [file ADHM-10-2101019-s001.pdf]

WILEY-VCH

© 2021 Wiley-VCH GmbH

ADVANCED HEALTHCARE MATERIALS

Supporting Information

for *Adv. Healthcare Mater.*, DOI: 10.1002/adhm.202101019

Manganese Ferrite Nanoparticles Encapsulated into Vitamin E / Sphingomyelin Nanoemulsions as Contrast Agents for High-Sensitive Magnetic Resonance Imaging

Sandra Díez-Villares,[†1,2,3]*, Miguel A. Ramos-Docampo,*[†4,5] *Andrés da Silva-Candal,*[6] *Pablo Hervella,*[6] *Abi J. Vázquez-Ríos,*[1,3]*Ana B. Dávila-Ibáñez,*[7] *Rafael López-López,*[3,7] *Ramón Iglesias-Rey,*[6] *Verónica Salgueiriño,**[4,5] *María de la Fuente**[1,3]

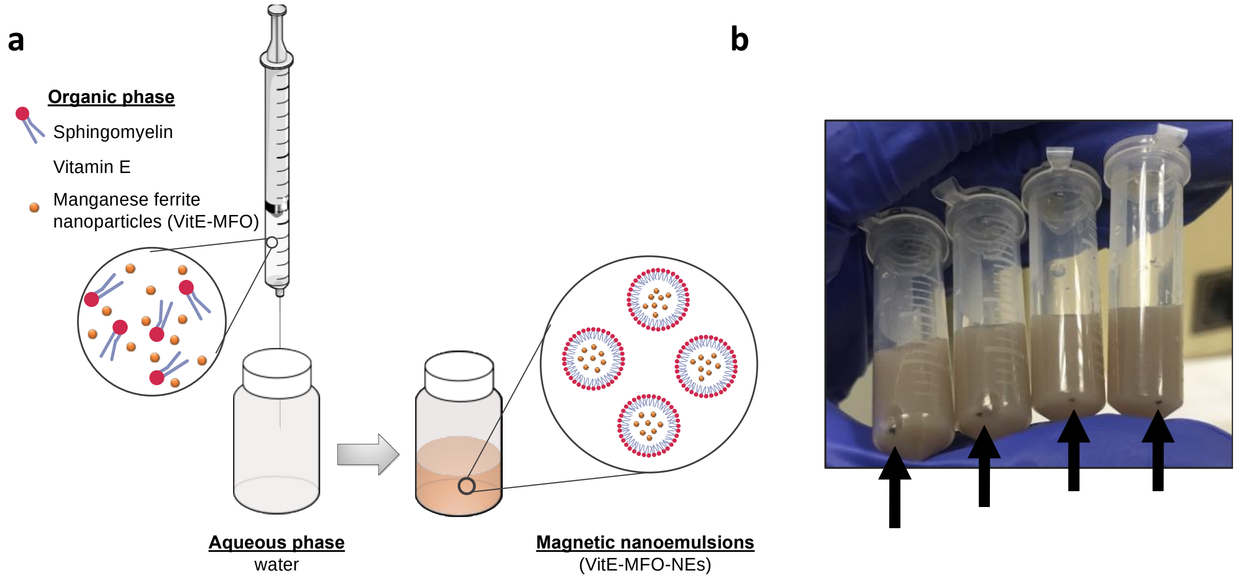

Figure S1. (a) Scheme of one-step ethanol injection method to obtain magnetic nanoemulsions and (b) photograph showing the VitE-MFO-NEs appearance and the pellet of non-encapsulated VitE-MFO after centrifugation.

ICP measurements.

VitE-MFO-NEs were prepared by ethanol injection and non-encapsulated magnetic nanoparticles were precipitated after centrifugation 10 min at 5.000 relative centrifugal forces

(rcf). Magnetic nanoemulsions were removed and the pellets were mixed with a dilution 1:2 nitric acid 65%:hydrogen peroxide 35%, following by microwave digestion. The resultant solution was diluted to 5 mL of final volume and analyzed by Optical emission spectrometry with inductively coupled plasma (ICP-OES).

Sample	Free MFO mass	Encapsulation yield (%)
1	2.0315 ± 0.0175	99, 14
2	1.7026 ± 0.0043	99,29
3	2.0759 ± 0,0299	99,13
4	3.8928 ± 0.0246	98,37
Average	2.4257 ± 0,8592	98,98

Table S1. ICP-OES quantification of the non-encapsulated magnetic nanoparticles.

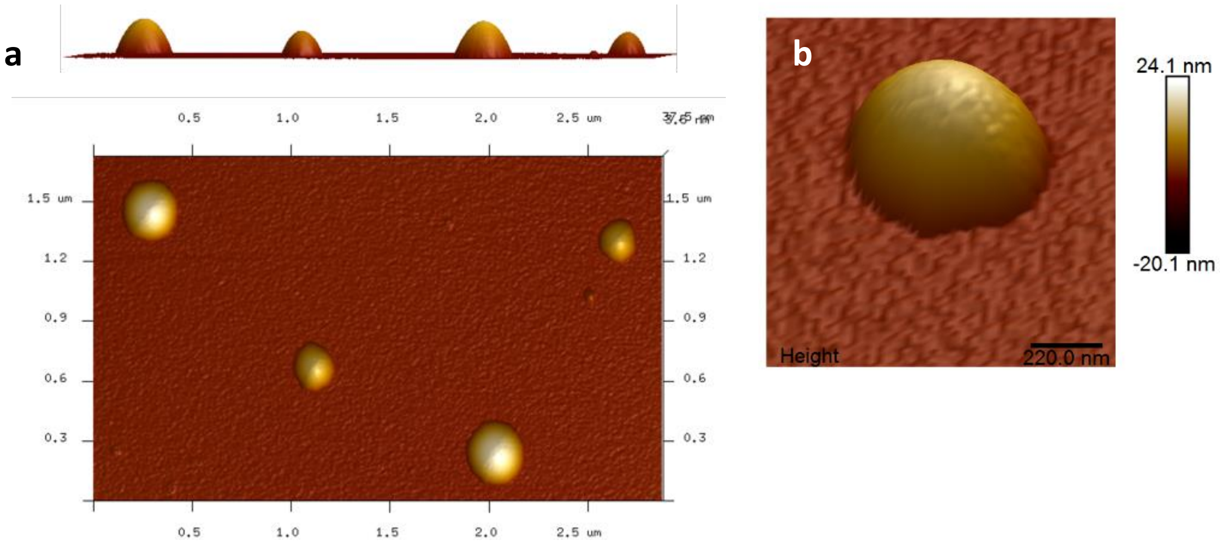

Figure S2. (a) AFM images and (b) 3D-high magnification image of the plain nanoemulsions (NEs).

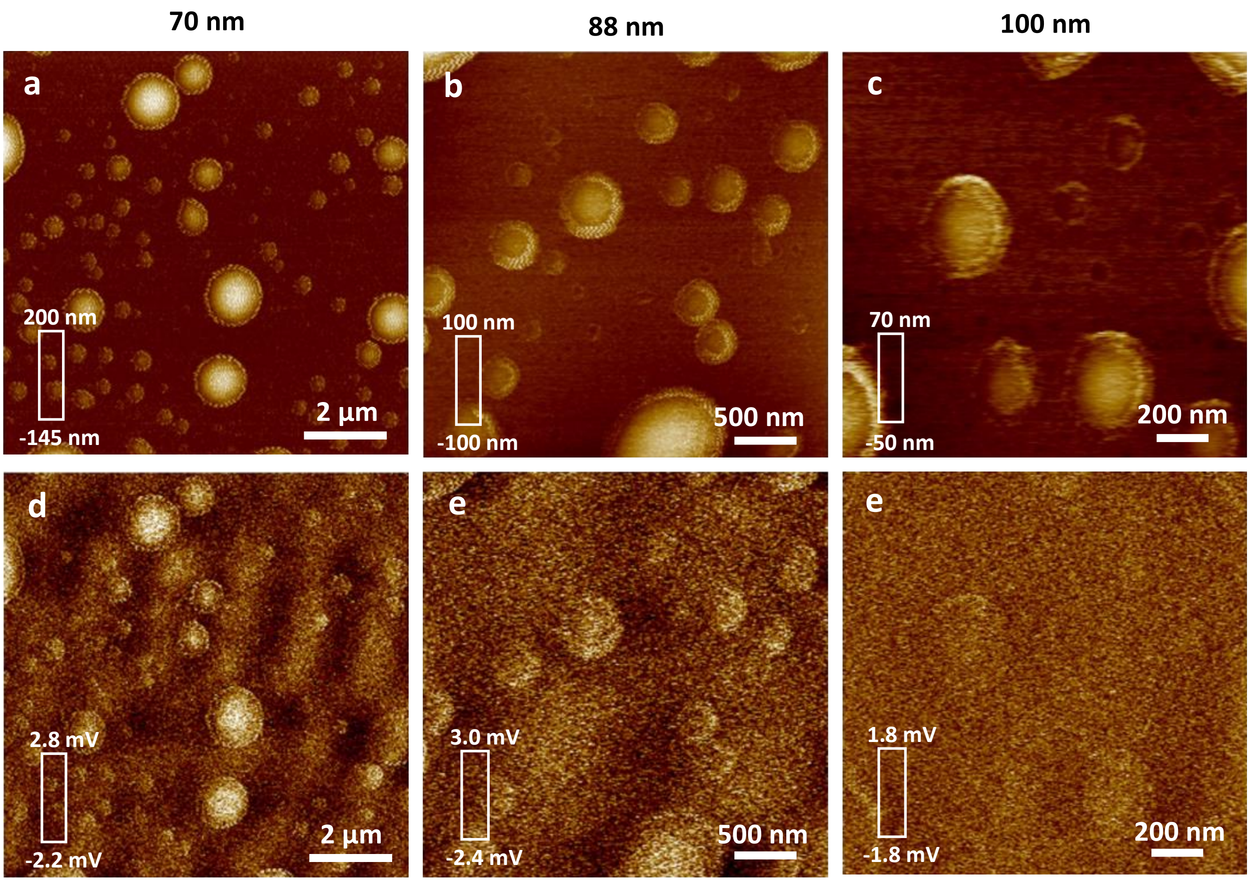

Figure S3. Phase contrast (a-c) and MFM (d-f) images of VitE-MFO-NEs scanning the same region at different lift heights (70, 88 and 100 nm) above the surface of the sample. MFM mode was operating in the presence of a 40-mT magnetic field.

In vitro MR imaging and longitudinal relaxivity.

To measure the longitudinal relaxivity of VitE-MFO-NEs, agar-based phantoms were prepared following the same procedure described in the manuscript and loaded with different concentrations of VitE-MFO-NEs (from 0.003 to 0.10 mM Fe). T_1-weighted images were acquired using RAREVTR sequence with 8.7 ms of echo time, 17.48 ms of effective echo time, 4 rare factor, 6 T_1 experiments (900, 2000, 3500, 5000, 7000, 11000 ms), 1 average, 1 repetition, flip angle of 180 °, 14 slices of 1 mm, with a field of view (FOV) of 7.5 cm × 7.5 cm (with saturation bands to suppress signal outside this FOV), and a matrix size of 300 × 300, giving an in-plane resolution of 250 µm/pixel and implemented without fat suppression. The relaxivity constant (r_1) was calculated as the slope of the curve obtained by fitting the T_1^{-1} values versus the Fe concentration in mM.

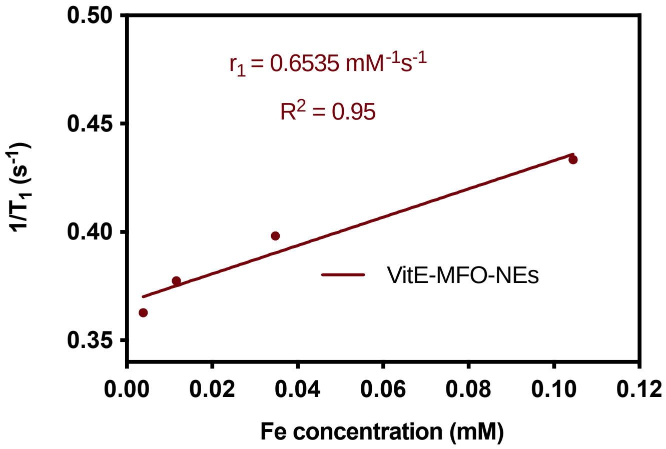

Figure S4. Longitudinal relaxivity (r_1) of VitE-MFO-NEs acquired with 9.4 T horizontal MR scanner.

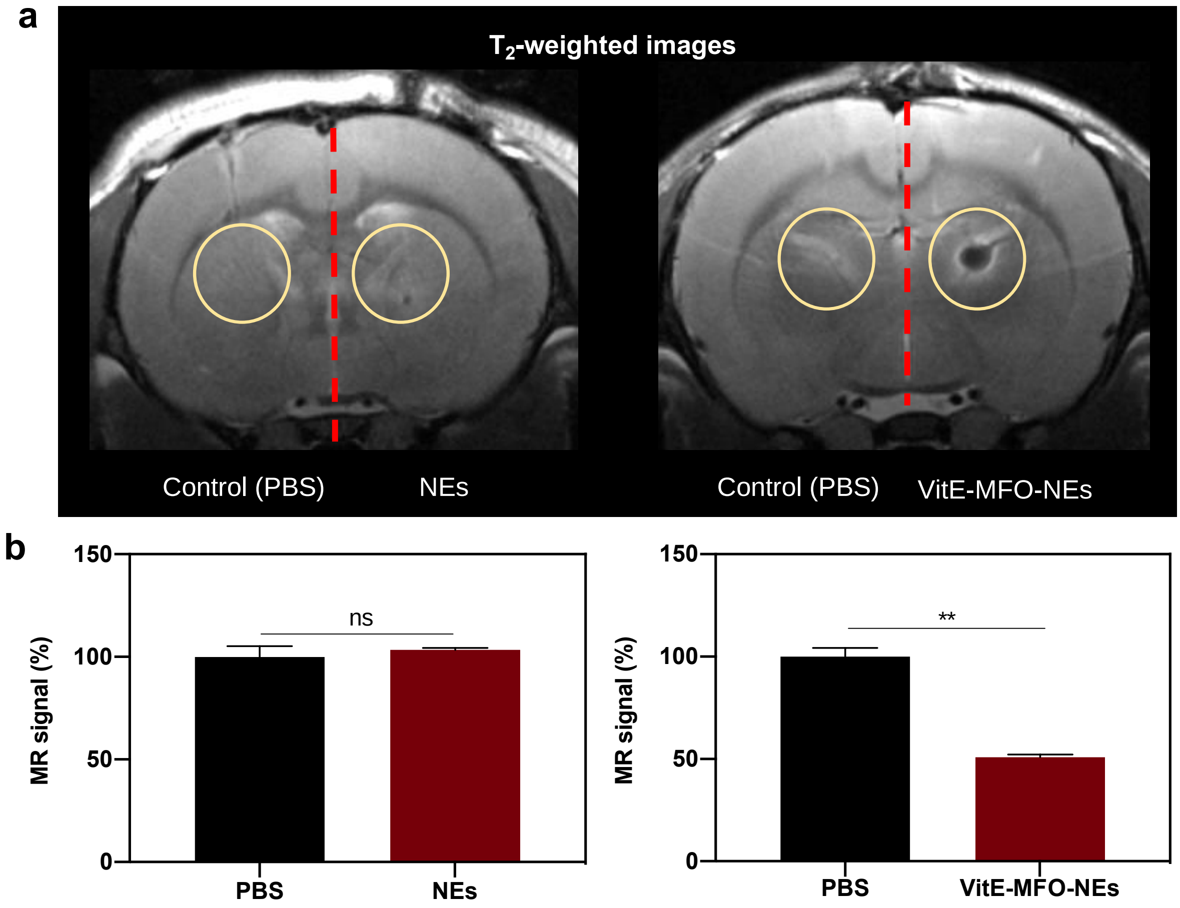

Figure S5. (a) T_2-weighted MR images of brain rats injected in the left cerebral hemisphere with PBS as a control and in the right hemisphere with the formulations NEs (image on the left) and VitE-MFO-NEs (image on the right). (b) Comparison of T_2-weighted MR signals between PBS and NEs (left) and PBS and VitE-MFO-NEs (right). MR signals of NEs and VitE-MFO-NEs are normalized to PBS as control (100 %). (n=3 per group) * ($p < 0.05$), ** ($0.05 > p < 0.001$), *** ($p < 0.0001$) was considered statistically significant.
